# Freshwater Foraminifera Biodiversity in New England (USA): Evaluation of Field Sites and a Botanical Garden

**DOI:** 10.1002/ece3.71557

**Published:** 2025-07-03

**Authors:** Adri K. Grow, Laura A. Katz

**Affiliations:** ^1^ Program in Organismic and Evolutionary Biology University of Massachusetts Amherst Amherst Massachusetts USA; ^2^ Department of Biological Sciences Smith College Northampton Massachusetts USA

**Keywords:** 18S rRNA, community analysis, low‐pH, metabarcoding, non‐marine, protists

## Abstract

Despite textbook definitions of foraminifera as marine organisms, foraminifera also occur in freshwater habitats, with first reports from ~150 years ago. Yet, to date, very few freshwater foraminifera have been described in part because these fragile organisms are rarely cultivable. There are fewer than 20 freshwater foraminifera species described both morphologically and molecularly, and a preliminary genome analysis has been done for only one species (*Reticulomyxa filosa*). In this study, we use foraminifera‐specific primers designed to amplify a portion of the 18S rRNA gene as a means of characterizing freshwater foraminifera community diversity from both field sites and a botanical garden. Our focus is on exploring trends in freshwater foraminifera composition in low‐pH bogs, carnivorous pitcher plants, and a greenhouse environment, with all sites sampled in Massachusetts and Maine (USA). Our preliminary results highlight the low diversity of freshwater foraminifera in these environments, which is in striking contrast to the tremendous diversity found among marine lineages. We also find lineages (i.e., operational taxonomic units [OTUs]) that are widespread and others that appear to be site‐specific, and we identify several clades of “unknown” freshwater foraminifera (i.e., those lacking previous molecular identification) that may be specific to the sampled locations. Together, these data help to expand our understanding of freshwater foraminifera biogeography through analyses of extreme habitats such as low‐pH bogs and fens.

## Graphical abstract

We explore trends in freshwater foraminifera richness and relative abundance across several low‐pH bog locations, including different ecological sites within each location, and from one established botanical garden habitat using a metabarcoding molecular technique. Our main findings confirm the relatively rare nature of freshwater foraminifera, and yet we find a remarkably broad phylogenetic diversity of foraminifera from these sites and determine that the majority of this molecular diversity was previously unknown. These data advance our understanding of the biology, ecology, and biogeography of freshwater foraminifera in New England, and specifically contribute to a broader understanding of their biodiversity and evolutionary history.

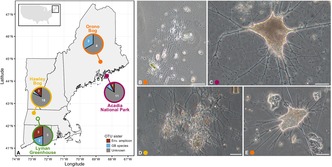

## 1 Introduction

Foraminifera are a diverse, more than 545‐million‐year‐old clade (Lee and Anderson 1991; Goldstein 1999; Bosak et al. 2012) of amoeboid single‐celled eukaryotes (i.e., protists). Characterized by their intricate spiderweb‐like network of pseudopods that stream cytoplasm bidirectionally, foraminifera are considered ecosystem engineers and contribute substantially to oceanic carbon cycling (Pinko et al. 2020; Kenigsberg et al. 2022). Despite textbook definitions of foraminifera as marine organisms, freshwater foraminifera were discovered over 150 years ago (Claparède and Lachmann 1859; reviewed in Holzmann et al. 2021). However, very few freshwater foraminifera have been described in part because these fragile organisms are rarely cultivable (Dellinger et al. 2014) and because they require lineage‐specific protocols for both microscopy and molecular work. To date, 18 freshwater foraminifera species have been described both morphologically and molecularly (Siemensma et al. 2021; Dellinger et al. 2014; reviewed in Holzmann et al. 2021; Siemensma and Holzmann 2023), and there is only one draft genome sequenced for a single species (Glöckner et al. 2014).

Due to fossilized marine foraminifera serving a critical role in reconstructing past climates and identifying oil deposits (Goldstein 1999; Musco et al. 2017; Taylor et al. 2018; Schwing et al. 2020; Alegret et al. 2021), research has been more concentrated on marine lineages, contributing to a bias in our biological understanding (Siemensma et al. 2021). As of 2020, there were 4932 foraminiferal genera defined in the World Registrar of Marine Species (WoRMS) and 55,884 species defined including extinct, extant, and infraspecies (Costello et al. 2013; Hayward et al. 2020). Of all foraminiferal binomial species, roughly 54,600 are accepted names and 9600 of them are extant (Hayward et al. 2020). This is in stark contrast to the 28 freshwater foraminifera species described to date, for which just over half have been defined using molecular tools in addition to the microscope (Holzmann et al. 2021; Siemensma and Holzmann 2023).

Freshwater foraminifera, when first discovered, were considered to belong to the genus *Gromia* (Dujardin 1841; reviewed in Holzmann et al. 2021), a lineage of Rhizaria sister to foraminifera. In light of molecular techniques, Pawlowski et al. (1999) provided evidence that the freshwater species *Reticulomyxa filosa* nested among marine foraminifera. With the continued addition of phylogenetic information of freshwater foraminifera, these lineages often fall among the monothalamids, a morphological description for foraminifera that have single‐chambered bodies. Despite this seemingly simple morphologic description, through the advent of molecular investigation (Pawlowski et al. 2013, 2014; Gooday et al. 2021; Holzmann et al. 2021; Sierra et al. 2022), monothalamid foraminifera are a non‐monophyletic group and serve as one of the more understudied but highly diverse groups of foraminifera. Five groups of freshwater foraminifera have emerged across the foraminifera phylogeny, indicating multiple independent origins of transition from marine to freshwater environments (Pawlowski et al. 2002; Holzmann et al. 2003, 2021; Siemensma et al. 2017). Given the limited molecular data available for freshwater foraminifera, compared to marine lineages, their phylogenetic placement continues to shift. One efficient way to explore freshwater foraminifera diversity is through the deployment of a metabarcoding approach (Pawlowski and Holzmann 2014; Pawlowski et al. 2016), which helps to understand the extent of biodiversity and establish a baseline that can be used for repeated assessment.

To characterize freshwater foraminifera biodiversity in New England, we deployed a metabarcoding study primarily focused in low‐pH bogs and in an established greenhouse environment. Bogs, environments that remain relatively stable over time as they accumulate peat through slowly decaying organic material, represent extreme environments that are highly acidic and nutrient‐poor (Gorham 1957; Clymo 1984; Blackford 2000). These environments, which serve as important global carbon sinks, are under threat by a changing planet (e.g., Ferretto et al. 2019). Given their usefulness as bioindicators in these distinct environments and their tremendous abundance, testate amoeba (Arcellinida) are often the target of diversity studies in these habitats (Lamentowicz and Mitchell 2005; Niedźwiecki et al. 2016; Ruggiero et al. 2020; Carballeira and Pontevedra‐Pombal 2021; Dufour et al. 2024). However, little is known about freshwater foraminifera in these environments, with only three described species having a type locality of *Sphagnum* moss from peat bogs to date (Siemensma et al. 2021; Siemensma and Holzmann 2023).

Here, we use amplicon primers developed to target a portion of the foraminiferal 18S rRNA gene (Thakur et al. 2022) on community RNA isolated from moss and carnivorous pitcher plants in low‐pH bog environments, as well as from biofilm (which we define here as the layer of all abiotic and biotic material attached to a surface) collected from two pools within a greenhouse. We assess the overall diversity of freshwater foraminifera in these sites, compare diversity across ecologically similar and distinct habitats, and discuss the generalist and specialist tendencies of lineages identified using our lab‐developed bioinformatic pipeline.

## 2 Materials and Methods

### 2.1 Sampling Site and Collection: Field Sites

We collected samples from two low‐pH bog environments in Maine (ME): Big Heath bog and Orono bog. In Acadia National Park, ME (referred to as Acadia going forward), we collected samples from Big Heath bog (permit ACAD‐2021‐SCI‐0040; 44.235817 N, 68.320533 W) and the Wonderland trail stream (44.2325 N, 68.318333 W, ~165 m from the Wonderland trailhead) on May 17th, 2021. In Orono, ME, we collected samples from the Orono Bog Boardwalk (44.868533 N, 68.725075 W) on May 19th, 2021 (Table 1). In both bogs, we collected *Sphagnum* spp. moss along an environmental gradient that spanned from the forested edge to the open center of the bog. In addition, in Acadia, we sampled biofilm and liquid from chambers of *Sarracenia purpurea* pitcher plants near the vegetative edge of the open bog and collected *Sphagnum* moss from either side of the Wonderland trail at the site of the stream. At all sites, we measured pH if there was sufficient moisture for the probe (Table 1 and Table S1).

**TABLE 1:** Summary of samples collected from freshwater sites in Maine (ME) and Massachusetts (MA) spanning a period from 2018 to 2023.

Date	State	Location	Site	Site type	Sample type	Volume (approx. mL)	No. of samples	pH
06/06/18^a^	MA	Hawley	Hawley Bog	Forest edge	*Sphagnum*	250	2	4–5
06/06/18^a^	MA	Hawley	Hawley Bog	Open bog	*Sphagnum*	250	6	4–5
05/17/21	ME	Acadia	Big Heath Bog	Forest edge	*Sphagnum*	30	1	NA
05/17/21	ME	Acadia	Big Heath Bog	Open bog	*Sphagnum*	30	1	3
05/17/21	ME	Acadia	Big Heath Bog	Pond edge	*Sphagnum*	30	2	3–4
05/17/21	ME	Acadia	Big Heath Bog	Pitcher plant	Swab + Fluid	5–13	6	4
05/17/21	ME	Acadia	Wonderland Trail	Stream	*Sphagnum*	30	2	2–3
05/19/21	ME	Orono	Orono Bog	Forest	*Sphagnum*	30	1	6
05/19/21	ME	Orono	Orono Bog	Forest edge	*Sphagnum*	30	1	NA
03/01/21	MA	Lyman	Warm Temp. house	Pool	Swab + fluid	15	2	NA
10/23/23	MA	Lyman	Warm Temp. house	Pool	Swab	0	4	6–7
10/23/23	MA	Lyman	Stove house	Pool	Swab	0	2	6

*Note:* We washed foraminifera cells off of *Sphagnum* spp. moss and used swabs to target the biofilm within *Sarracenia purpurea* pitchers and along the walls of the greenhouse pools. Number of samples refers to the number of successful PCR reactions for pooled samples (where applicable, see Table S1). Approximate ranges for pH are provided based on in situ measurements when conditions allowed; however, near the forest and forest edge, measurements could sometimes not be obtained due to a lack of moisture (see Table S7 for details).

Abbreviation: Temp., temperate.

^a^ Details on samples in Ruggiero et al. (2020).

For each moss sample, we placed a few strands of moss in a sterile 50 mL conical tube with ~35 mL of Volvic Natural Mineral (Danone) or 2 μm filtered in situ water and gently mixed by inversion to dislodge cells from the moss. These samples were then pre‐filtered through a 300 μm mesh filter to remove large debris and serially filtered through an 80, 10, and 2 μm Nylon Net or Isopore PC Membrane Filter (MilliporeSigma) using vacuum filtration. For each pitcher plant sampled, we swabbed the inside of the pitcher with a sterile cotton‐tip applicator and collected 5–15 mL of fluid using a sterile transfer pipette. The cotton‐tip applicator was added to the fluid sample and mixed thoroughly. We then pre‐filtered each pitcher sample on a 300 μm mesh filter and directly filtered on a 2 μm Isopore PC Membrane Filter using vacuum filtration. Each filter obtained was placed in a 1.5 mL tube prepared with 500 μL of Buffer RLT (Qiagen) and stored at −80°C prior to total RNA extraction.

In addition to the two field sites described above, a historical set of samples (Ruggiero et al. 2020) collected on June 6th, 2018 from Hawley Bog (42.575686 N, 72.890606 W) in Hawley, MA was added to this study as well. At this site, *Sphagnum* spp. moss samples were collected along two transects 26 m apart; four sites were sampled along each transect approximately 8 m apart that spanned a gradient from the forested edge of the bog towards the center (see Ruggiero et al. 2020). For each Hawley sample, 50 g of moss was washed with 250 mL of 0.2 μm filtered in situ water, prefiltered with a 300 μm mesh filter to remove large debris, and serially filtered through a 100, 35, 10, and 2 μm filter using gravity (100 and 35 μm) and vacuum filtration (10 and 2 μm). All filters were placed in 1.5 mL tubes prepared with 1 mL of Buffer RLT and stored at −80°C prior to total RNA extraction (see Ruggiero et al. 2020 for more detailed information).

### 2.2 Sampling Site and Collection: Botanical Garden Sites

At the Smith College Lyman Plant House and Conservatory (42.318903 N, 72.640044 W), we sampled small freshwater pools from the Stove house and the Warm Temperate house on March 1, 2021 and October 23, 2023 (Table 1; Table S1). The Warm Temperate house is a greenhouse with various types of tropical and subtropical plants, including numerous carnivorous plants. Pitcher plants (*Nepenthes* spp.) are propped above a 2.5 × 2.5 m concrete pool containing duckweed ( *Lemna minor* ) and giant salvinia ( *Salvinia molesta* ) separated by a wooden wall (Figure S1). In 2021, we swabbed only the wood wall of this pool for both plant‐dominated sections using a sterile cotton‐tip applicator targeting a 25 cm^2^ area approximately 10 cm below the surface of the water. We also used a transfer pipette to scrape the wall and aspirate 15 mL of water for each of the swab samples. The cotton‐tip applicator was then added to the fluid sample and mixed thoroughly before pre‐filtering through a 780 μm mesh filter and then filtering on a 2 μm Isopore PC Membrane Filter (MilliporeSigma) using vacuum filtration. Filters were placed in 1.5 mL tubes prepared with 500 μL of Buffer RLT (Qiagen). In 2023, we used a sterile cotton‐tip applicator to swab a 25 cm^2^ area of the walls approximately 10 cm below the surface of the water from both the concrete and wooden walls for each plant‐dominated section of the pool. For these swab‐only samples, we simply cut the cotton tip into 1.5 mL tubes prepared with 500 μL of Buffer RLT.

In regard to the Stove house, this is a greenhouse with various species of orchids as well as plants belonging to the Araceae family propped above a 2.5 × 6.5 m pool housing various marginal and economically important potted plants (Figure S1). On the same day in 2023 as above, we used a sterile cotton‐tip applicator to swab a 25 cm^2^ area of the concrete walls approximately 10 cm below the surface of the water on each of the longest sides of the pool directly opposite one another. We cut the cotton tip end of the swabs into 1.5 mL tubes prepared with 500 μL of Buffer RLT for each sample. All greenhouse samples were stored at −80°C prior to total RNA extraction.

### 2.3 Community RNA Extraction

We used the RNeasy Mini Kit (Qiagen) to extract community RNA following the manufacturer's protocol. We specifically extracted community RNA, instead of DNA, to target the active community present at the time of sampling. RNA was processed further to remove DNA using the TURBO DNA‐*free* Kit (Invitrogen) followed by the SuperScript III First‐Strand Synthesis System (Invitrogen) with Random Hexamers (ThermoFisher Scientific) to create single‐stranded cDNA as described in Sisson et al. (2018) and Ruggiero et al. (2020). The resulting cDNA was stored at −80°C prior to PCR amplification.

### 2.4 Amplification and Sequencing

We used foraminifera‐specific primers (Thakur et al. 2022) to amplify a ~250 bp hypervariable region of the small subunit ribosomal RNA (SSU‐rRNA) gene following protocols detailed in Thakur et al. (2022). We performed PCRs in triplicate and pooled these together to reduce PCR bias (Sisson et al. 2018; Lahr and Katz 2009; Jung et al. 2012). Sequence libraries were prepared by the University of Rhode Island RI INBRE Molecular Informatics Core for Illumina MiSeq High Throughput Sequencing.

### 2.5 Bioinformatic Analysis and Data Curation

We describe our bioinformatic processing in detail here; however, we want to emphasize that the operational taxonomic units (OTUs) we discuss in this study result from hand‐curation to remove marine contaminants that resulted from other studies in our lab (e.g., based on visual inspection of alignments and trees, by BLAST information); we believe our final list of OTUs is conservative and appropriate given the limited state of knowledge for this group of foraminifera.

Following pilot analyses that revealed low diversity, we decided to combine size‐fractioned samples by pooling raw reads for samples that were originally serially filtered (Table S1). For each sample, we took the resulting raw reads and created OTU libraries using the bioinformatic pipeline outlined in Sisson et al. (2018). To summarize the initial steps: trimmed raw reads were merged into paired‐end reads using PEAR (Zhang et al. 2014) and OTUs were called using default SWARM (Mahé et al. 2015). Built‐in quality checks of the pipeline remove OTUs that had one read, that were chimeric, or that were less than 65% similar to the most abundant OTU (i.e., highly divergent; see Sisson et al. 2018 for details). After this initial automated curation, we used a phylogenetic approach with a hand‐curated database of 3403 full‐length SSU‐rRNA reference sequences from GenBank that belong to the major clade SAR (Stramenopila, Alveolata, Rhizaria) and 323 outgroup sequences (i.e., Amoebozoa, Archaeplastida, Bacteria, Excavata, Opisthokonta, and some Orphan lineages) to determine OTU placement. We identified 27 OTUs that fell among the outgroup to all of SAR to remove from further analysis, leaving 127 OTUs that fell within SAR (File S1), of which only 68 fell within the Foraminifera clade of Rhizaria (Table S2). We then removed the 59 OTUs that fell outside the foraminifera clade from further analysis (Table S2).

To further refine the 68 foraminiferal OTUs, we first built an unrarefied OTU table (Table S3) through the pipeline and set several curation parameters based on non‐rarefied reads. At this stage, we removed any sample with fewer than 500 reads (i.e., likely failed samples) and removed any OTU with less than 100 unrarefied reads or with only one occurrence. This step removed 20 samples and 18 OTUs; however, it is important to note that these OTUs could be freshwater foraminifera OTUs, but that this approach conservatively estimates the diversity described by our data. As a result, an additional two samples with fewer than 500 reads were removed. Then, we used MAFFT L‐INS‐i (Katoh and Standley 2013) to align the remaining 50 OTUs with a hand‐curated foraminifera reference containing 18 freshwater foraminifera species from GenBank, 13 environmental sequences (from GenBank plus a subset of OTUs in Thakur et al. 2022), and 17 other full‐length monothalamid species from GenBank. Information on all references used to build the phylogenetic trees can be found in Table S4. Gaps in alignments were masked at several different percentages (e.g., 90, 95, 99) to assess how masking impacted relationships in phylogenetic trees that were built using RAxML with the GTRGAMMAI model (Stamatakis 2014), and overall, we saw similar phylogenetic relationships except for the OTUs with no close relatives, which shifted in different iterations as expected. From phylogenetic assessment and also by NCBI BLAST, we identified six OTUs to remove that were closely related to marine foraminifera and were likely contaminants. We also noticed one sample that we removed from further analysis because it was highly contaminated by several marine OTUs. We then ensured that the final unrarefied table met our original curation parameters (samples must have more than 500 reads, OTUs must have at least 100 reads, OTUs must also have more than one occurrence) before moving forward to rarefying the samples (all information for samples and OTUs removed can be found in Tables S2 and S5).

Following the above unrarefied curation steps, we settled on a final list of 30 samples and 42 putative freshwater foraminifera OTUs. We then rarefied our samples, given that we had a large discrepancy between our smallest (714 reads) and our largest sample (294,449 reads) and as an effort to normalize the data. All samples were rarefied at 10,000 reads, with seven of the 30 samples having less than 10,000 reads (i.e., all reads in these samples were considered moving forward), and a final rarefied OTU table was created (see Tables S6 and S7). To account for index switching (Costello et al. 2018; MacConaill et al. 2018), we adjusted rarefied reads in the following manner: (1) for any OTU with more than 1000 reads, for any occurrence in a given sample with fewer than 20 reads, we replaced the read number with a zero; (2) for any OTU with fewer than 1000 reads, for any occurrence in a given sample that was one read, we replaced the read number with a zero. Finally, as a last curation step, we only considered OTUs with more than 20 total rarefied reads for the final analyses—four OTUs were removed at this step (Table S2).

The above detailed hand‐curation steps resulted in a final number of 30 samples and a final list of 38 putative freshwater foraminifera OTUs (Table S8). The final phylogenetic tree was constructed from a 90% masked gap MAFFT L‐INS‐i alignment using RAxML with the GTRGAMMAI model. To estimate support for the tree topology, we conducted rapid bootstrap analysis with 1000 bootstrap replicates and labeled nodes with greater than 80% bootstrap support (see also File S2). We calculated dissimilarity matrices with weighted UniFrac distance and generated principal coordinate analyses (PCoAs) using the R package phyloseq (McMurdie and Holmes 2013). To discuss OTU identity moving forward, we rely most heavily on phylogenetic placement among freshwater foraminifera references and published environmental sequences, as BLAST results rarely had robust hits to the data currently available on GenBank. In addition, based on inspection of the final tree topology, we categorize OTUs into one of three categories: (1) as falling sister to environmental freshwater foraminifera sequences, (2) as falling sister to a GenBank freshwater foraminifera species, or (3) as an unknown lineage (i.e., lacking molecular data at the time of our analyses).

## 3 Results

### 3.1 Metabarcoding Summary

After careful curation detailed above in the methods, we identified a conservative set of 38 putative freshwater foraminiferal OTUs represented by 249,659 rarefied reads. We believe the extensive hand curation here is warranted given the apparent low abundance and low diversity of freshwater foraminifera in our samples, coupled with the limited number of reference sequences available for these lineages. We also emphasize that we present a conservative estimate of 38 OTUs identified in our samples as we had to contend with an unusually high amount of contamination by ciliates, by marine OTUs introduced from concurrent studies in the lab, and by PCR artifacts—we believe these problems arose given the relative rarity of freshwater foraminifera in the habitats sampled.

**FIGURE 1:**
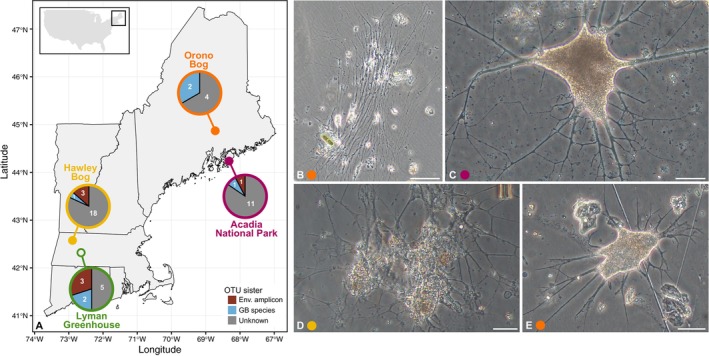
(A) Map of New England (northeast United States) showing the four major sampling locations of this study. We sampled three field sites: Big Heath bog and Wonderland stream in Acadia National Park, ME, Orono Bog in Orono, ME, and Hawley bog in Hawley, MA. In addition, we sampled from one greenhouse environment, the Lyman Plant House and Conservatory located in Northampton, MA (see Table 1 for more detail). For each location, a pie chart depicts the number of freshwater foraminiferal OTUs that were found at that site, separated by their sister relationship based primarily on inferred phylogenetic sister relationships (see Figure 2). Env. = environmental; GB = GenBank; MA = Massachusetts; ME = Maine. (B–E) Exemplar images of freshwater foraminifera found from *Sphagnum* spp. moss samples from low‐pH bogs. (B, E) Collected from *Sphagnum* at the forested edge of Orono bog. (C) Collected from *Sphagnum* at the edge of Big Heath bog. (D) Collected from *Sphagnum* at the forested edge of Hawley bog. Scale bars: B = 100 μm; C–E = 50 μm.

The inferred 38 OTUs come from 30 samples collected from three low‐pH bogs sampled in 2018 and 2021, and one greenhouse environment sampled in 2021 and 2023 (Figure 1A; Table 1). From each of the three field sites, we observed and photo‐documented several freshwater foraminifera that serve as examples of the types of potential morphologies among the OTUs we identified using a metabarcoding approach (Figure 1B–E). The top five OTUs (OTU1, OTU2, OTU3, OTU6, OTU7) account for 72.9% of all curated reads, with OTU1 representing more than a quarter, 34.2%, of all curated reads. The top 10 OTUs (including OTU5, OTU16, OTU17, OTU32, OTU45) account for nearly all reads in the study at 95.1%, indicating relatively low diversity given the sites we sampled and our sequencing effort. Overall, we found that the field sites harbor the most OTU richness (2–15 OTUs per sample), the majority of which we infer to be unknown (i.e., no known close relatives; Figure 1).

### 3.2 Freshwater Foraminifera Diversity From Four Locations in New England

**FIGURE 2:**
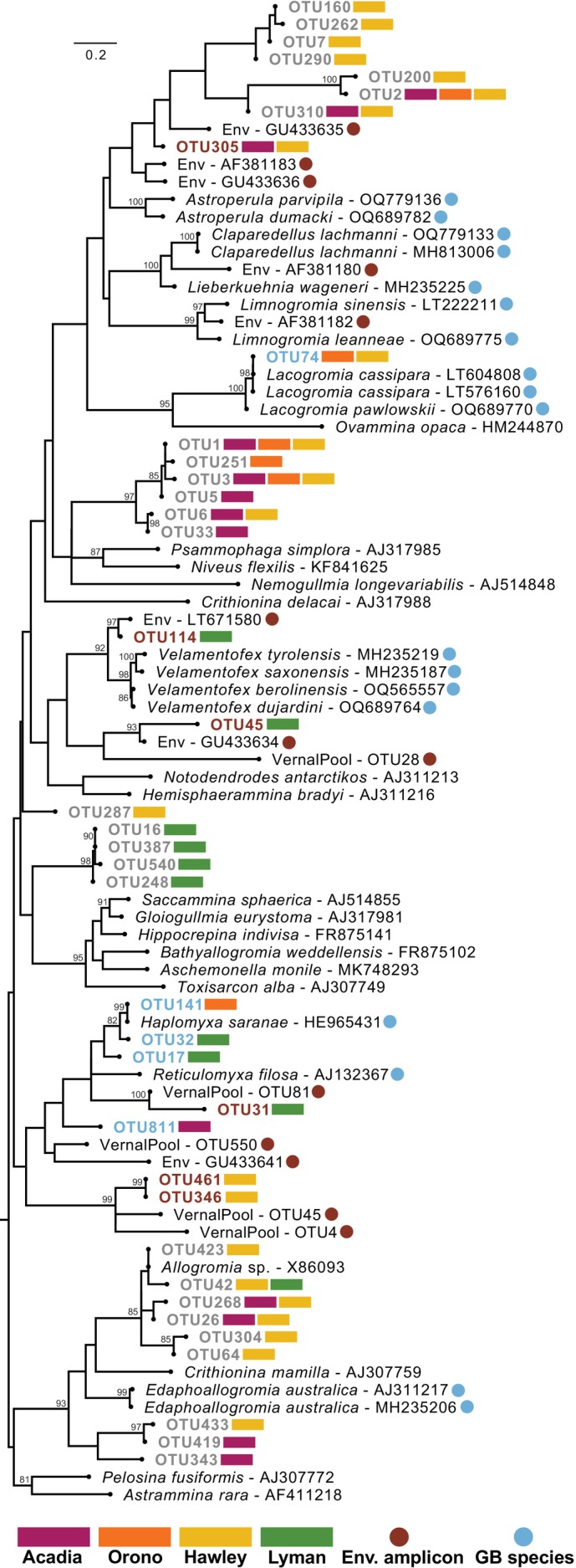
Phylogenetic tree showing the relationship of 38 putative freshwater foraminifera OTUs among 18 freshwater foraminifera (blue circles) and 10 environmental (brown circles) references (nested among 17 other monothalamid foraminifera. OTU labels are colored by our inferred sister relationships for that OTU based primarily on phylogenetic relationships. Shaded rectangles indicate the location(s) where each OTU was detected. We conducted rapid bootstrap analysis with 1000 bootstrap replicates and labeled nodes with greater than 80% bootstrap support. See File S2 for all bootstrap node information and Table S4 for details on all references included. Env. = environmental; GB = GenBank; OTU = operational taxonomic unit.

For phylogenetic analysis, we combined our 38 inferred OTUs with a set of 18 reference freshwater foraminifera species, 13 freshwater environmental sequences, and a subsampled set of sequences from marine monothalamid species (Table S4) that represents only a small portion of all foraminiferal diversity (see Figure S2). Five of our 38 OTUs phylogenetically nest among the few morphologically and molecularly described freshwater species, six OTUs nest among the environmental sequences, and the remaining 27 OTUs have no close relatives and often form their own clades (Figure 2). Overall, Hawley was the most diverse site with 22 OTUs identified in this location, whereas Acadia and Orono have 13 and six OTUs identified, respectively.

Only two of our 38 OTUs in the whole study are identical (based on alignment inspection and BLAST) to previously characterized species of freshwater foraminifera: OTU74 is identical to the agglutinated *Lacogromia cassipara* (GenBank numbers LT604808; LT576160) while OTU141 is identical to the naked (i.e., non‐testate) *Haplomyxa saranae* (GenBank number HE965431). This latter species is morphologically similar to the few cells we were able to observe from these field sites (Figure 1B–E). Of the five OTUs that are sister to described freshwater foraminifera, apart from the two just mentioned for which we have a clear identity, the other three OTUs nest within the Reticulomyxidae family (Figure 2; genera *Haplomyxa* and *Reticulomyxa*). As for the six OTUs that are sister to environmental sequences, OTU31, OTU346, and OTU461 are most similar to OTUs isolated from vernal pool sediments sampled in Massachusetts and previously characterized in our lab (Figure 2, Table S4). Two other OTUs sister to environmental sequences (OTU45 and OTU114) are most similar to environmental sequences isolated from sediments in France and Switzerland (Figure 2, Table S4). On the other hand, OTU305 nests within a large clade containing a few environmental sequences (and more distantly related unknown OTUs, see below) that are sister to robust clades of known freshwater foraminifera (e.g., the genera *Claparedellus*, *Lieberkuehnia*, and *Astroperula*). Within this territory for OTU305 as well are several environmental sequences, some of which were collected in sediments from the Hudson River and Washington Park Lake both in Albany, NY (GenBank numbers AF381180, AF381182, and AF381183; Figure 2).

The remaining “unknown” (i.e., unable to confidently assign identity) 27 OTUs form ~four distinct clades plus one single branch, all of which do not have any close known relatives (Figure 2). For example, we see a location‐specific clade of OTU16, OTU248, OTU387, and OTU540 that were all found within the Lyman greenhouse samples, which form their own clade off on a long branch sister to marine lineages. In addition, a field site‐specific clade of OTUs containing OTU1 (the most relatively abundant OTU in the study) is comprised of six OTUs in total, with all three field sites represented, and no phylogenetically close relatives ground this clade among other freshwater foraminifera. Similarly, there is an unknown clade of four OTUs (OTU7, OTU160, OTU262, and OTU290) that were only found within samples from the Hawley field site. These four OTUs are sister to other unknown OTUs and more distantly related to one environmental sequence. In addition, although there is a clade of six OTUs most closely related to an *Allogromia* sp. (GenBank number X86093), we label these OTUs as unknown because these sequences lack a substantial portion of the region we amplified by PCR and represent only small fragments (all being 107 bp in length).

### 3.3 Freshwater Foraminifera Community Patterns From the Field and a Botanical Garden

**FIGURE 3:**
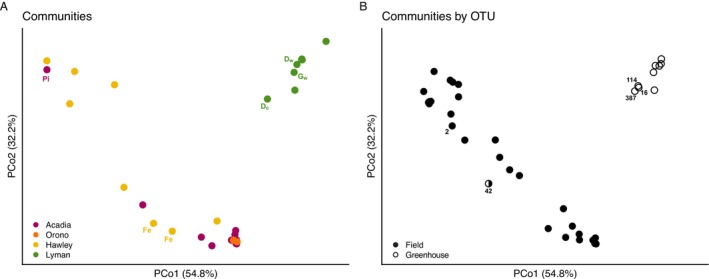
(A) Principal coordinate analysis (PCoA) with weighted (accounting for relative abundance) UniFrac distance (accounting for phylogenetic relationships) describes 87.0% of variance among samples from the four sampling locations. (B) OTUs that underlie the samples are arrayed in the same ordination space. Open circles represent the nine OTUs that are only present in the greenhouse samples. Exemplar samples and OTUs are labeled and discussed in the main text. Acadia, Orono, and Hawley = field locations; D = duckweed; Fe = Forest edge; = giant salvinia; Lyman = greenhouse location; OTU = operational taxonomic unit; Pi = Pitcher plant; subscript c = concrete wall; subscript w = wooden wall.

PCoA with weighted UniFrac distance (accounting for both relative abundance and phylogenetic distance) reveals that the greatest diversity among samples was found at Hawley bog (Figure 3A). Within Hawley, there is no distinct clustering among the six different open bog samples, suggesting that the community assemblages were quite different despite their general proximity to one another (transects 26 m apart, sites within each transect 8 m apart). This is best seen by looking at the Hawley samples spread across PCo1 and PCo2 that each respectively represent 54.8% and 32.2% of the variation among all samples (Figure 3A). However, the two forest edge samples from Hawley are more similar to one another, suggesting that these two sites 26 m apart along the forested edge may harbor similar assemblages of freshwater foraminifera. For the other two field sites, located ~78 km apart in Maine, two Orono samples and eight of the 12 Acadia samples cluster tightly together in ordination space, suggesting that the communities from these two field sites are similar. However, four samples from Acadia are more distinct, with one pitcher plant sample in particular in the top left corner of ordination space (Figure 3A) completely distinct from all other pitcher samples as well as all other Acadia samples.

We also observe some variation among the eight greenhouse samples, and no apparent clustering by the two different pool types. For samples that were taken within the same small pool, we do not observe similarity among these samples and instead find that these samples are distinct from one another (Figure 3A). Here, it does seem that there may be a distinction between samples taken from the wooden (subscript *w*) versus concrete (subscript *c*) walls of the same pool (Figure 3A). However, the limited sampling size in the greenhouse prevents a strong conclusion.

Of note, the field and greenhouse samples do not overlap in ordination space (Figure 3A), nor do the 38 OTUs characterized from within these samples (Figure 3B). Open circles indicate the nine OTUs that are only found in the greenhouse samples (Figure 3B) with OTU16, OTU114, and OTU387 being drivers of the leftmost greenhouse samples (Figure 3A). While OTU42 was found in both a field and greenhouse sample, given where it is in ordination space suggests that it has a stronger influence in the field sample (696 reads) as opposed to the greenhouse sample (21 reads). For the pitcher plant sample with the greatest difference from all other Acadia pitcher plant samples (Figure 3A, top left corner), OTU2 acts as a major influence in this specific sample and also in the distinction seen between this and other samples in the upper left corner of the plot (Figure 3A,B). OTU2, in an unknown clade distantly related to an environmental sequence (Figure 2), has 9685 reads in this single pitcher sample while its only other occurrence in a pitcher plant sample is represented by 181 reads. In addition, this distinct pitcher sample is most similar to one open bog sample from Hawley in which OTU2 has 9412 reads in that sample alone. This indicates that the relatively increased abundance of OTU2 in these samples is driving the major difference between these two samples from their other ecologically similar samples. In addition, the next two closest samples in ordination space are Hawley open bog samples that also had a relatively increased abundance of OTU2. Overall, these community patterns observed by PCoA highlight the lack of strong assemblage by general sampling location, prompting a closer examination of occurrence and relative abundance of individual lineages across the different site habitats within each sampling location (see below).

### 3.4 Habitat Specificity Among Freshwater Foraminifera OTUs

**FIGURE 4:**
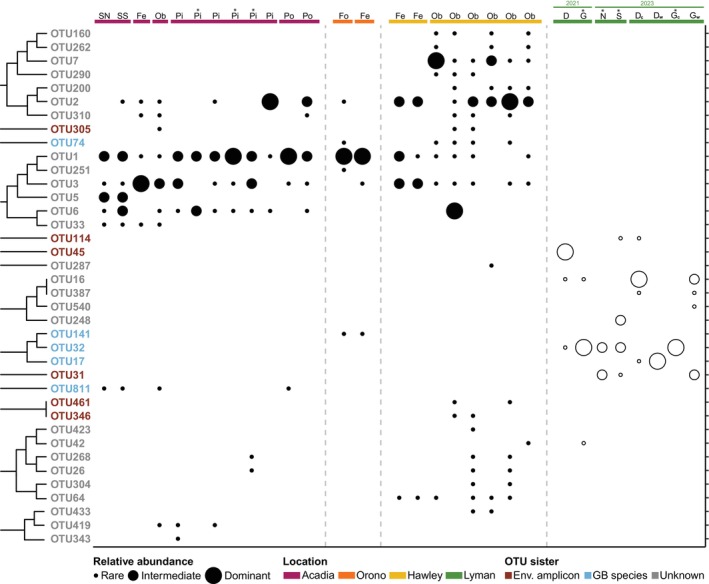
Ecological distribution of the 38 OTUs by clade (from top to bottom as ordered in Figure 2) in 22 field (filled circles) and eight greenhouse (open circles) samples based on relative abundance. For any given site, read cutoffs were used to determine whether the OTUs presence was rare, intermediate, or dominant in each sample. An OTUs presence is “rare” if its reads represent less than 25% of all reads for a given sample, an OTUs presence is “intermediate” if its reads represent between 25% and 75% of all reads for a given sample, and an OTUs presence is “dominant” if its reads represent more than 75% of all reads for a given sample. Asterisks indicate the samples that had fewer than 10,000 reads and therefore were not actually rarefied. See Table S7 for detailed information on the samples listed from left to right at the top of this figure. D = duckweed; Fe = forest edge; Fo = forest; G = giant salvinia; *N* = stove house pool north; Ob = open bog; OTU = operational taxonomic unit; Pi = pitcher plant; Po = pond; S = stove house pool south; SN = wonderland stream north; SS = wonderland stream south; subscript *c* = concrete wall; subscript *w* = wooden wall.

Overall, our data indicate that some OTUs may be generalists while others may be considered more specialized. For example, OTUs identified in the greenhouse were only detected from greenhouse samples and not any of the field samples (Figure 4) with only one exception. This exception is OTU42, the only OTU with a signal in a field and greenhouse site, with 21 of its 717 reads in the greenhouse. However, OTU42 is in the clade sister to *Allogromia* sp. (Figure 2), and as mentioned earlier, should be evaluated with caution as this OTU is represented only by small fragments of rDNA. Although we have few greenhouse samples, we did find some evidence of continuity within the greenhouse across sampling years. OTU16 (unknown) and OTU32 (sister to *H. saranae*) were detected in samples collected in both 2021 and 2023 (Figure 4), more than two years apart, suggesting that these two lineages may be well established in the greenhouse environment. Beyond this, our greenhouse samples are limited, and more sampling is needed to infer patterns between the two pools or between the different substrates sampled.

Among our field samples, several OTUs are nearly ubiquitous across the three sampling locations (e.g., OTU1, OTU2, and OTU3; Figure 4). However, several OTUs are specific to only one sampling location; for example, Hawley has the most OTUs present within a location (22 OTUs) with 12 of those 22 being specific to Hawley alone. Additionally, we can draw patterns of within‐location patterns exemplified by 18 of the 22 OTUs in Hawley that are only found in the open bog sites and not present in either of the forest edge sites (Figure 4). We observed a similar pattern at the Wonderland stream in Acadia where OTU5 (an unknown ) appears specific to this location and particular site (Figure 4), a site where the pH was especially low (pH 2–3; Table 1). The same is true for OTU141 (likely *H. saranae*) and OTU251 (unknown), which are the only OTUs specific to the Orono location. Although we see several cases that highlight the specificity among these lineages, including both between and within sampling locations, overall, we see that freshwater foraminifera distribution and abundance remains patchy in this system and given our sampling.

## 4 Discussion

We analyzed amplicons generated with foraminifera‐specific primers to characterize the diversity of freshwater foraminifera from three low‐pH bogs and one greenhouse environment, all located in the northeast United States (Table 1, Figure 1). Across collections from *Sphagnum* spp. moss and a few *S. purpurea* pitcher plants in field sites as well as two small pools in a greenhouse site, we find: (1) low OTU richness but broad phylogenetic diversity of freshwater foraminifera in these habitats, (2) a number of unknown lineages specific to each location, and (3) evidence of ecological partitioning of both known and unknown freshwater foraminifera lineages.

### 4.1 Inferences of Freshwater Foraminifera Richness

Our finding of 38 curated freshwater foraminifera OTUs (Figure 2) is consistent with the literature on non‐marine foraminifera species richness. Freshwater foraminifera are considered relatively rare and exhibit low species richness (Pawlowski and Holzmann 2002; Holzmann et al. 2021; Siemensma et al. 2021; Siemensma and Holzmann 2023), historically being found haphazardly and are oftentimes discovered as bycatch in samples collected for other purposes (Figure 1; Holzmann et al. 2021; Siemensma et al. 2021; Siemensma and Holzmann 2023). In fact, our own experiences support the rarity of these lineages, at least in low‐pH bogs and fens, as we have only seen a handful of cells (e.g., Figure 1) despite hundreds of hours of observations for Arcellinida within samples from these same sites (Ruggiero et al. 2020; Weiner et al. 2022, 2023; Dufour et al. 2024). In targeted eDNA (environmental DNA) studies from environments that span soil, river, and lake samples, no more than 50 sequences (amplicon and PCR/cloning) have been attributed to freshwater foraminifera lineages in a given study (Holzmann and Pawlowski 2002; Holzmann et al. 2003; Lejzerowicz et al. 2010; Thakur et al. 2022). For the majority of our curated OTUs, we were unable to assign any family, genera, or species identity (Figure 2), a common theme in protist metabarcoding studies in which typically about one‐third of taxa identified cannot be assigned to a known lineage (reviewed in Burki et al. 2021).

The low diversity of freshwater foraminifera in this study contrasts with both amplicon and morphological analyses of such lineages in marine settings. Studies in marine environments find hundreds of foraminifera lineages (e.g., OTUs, Amplicon Sequence Variants [ASVs]; both of which are proxies for species) from habitats that span from the deep sea to coastal mudflats (Laroche et al. 2016; Morard et al. 2018; Nguyen et al. 2023; Li et al. 2023; Singer et al. 2023; Girard et al. 2024). With the same primers as used in this study, we detected more than 500 amplicon sequences from marine samples collected from sites in Guam, Palau, and New England (Thakur et al. 2022). Among protist groups in general, hundreds to thousands of lineages are being detected per metabarcoding study (Gran‐Stadniczeñko et al. 2019; Grattepanche and Katz 2020; Ruggiero et al. 2020; Egge et al. 2021; Sleith and Katz 2022; Grow et al. 2023), highlighting just how ubiquitous microeukaryotes are in nature. Freshwater foraminifera, on the other hand, may have relatively low richness and abundance, perhaps due to the physiological barriers marine lineages face to successfully invade freshwater habitats (e.g., osmoregulation; Iglikowska and Pawłowska 2015; Annenkova et al. 2020; Holzmann et al. 2021). This barrier has also been described for dinoflagellates, another microeukaryotic clade in which the majority of their diversity is marine with far fewer freshwater representatives (Taylor et al. 2008; Mertens et al. 2012). However, the difference in diversity between freshwater and marine species is much greater for foraminifera than for dinoflagellates.

### 4.2 Phylogenetic Relationships Among Freshwater Foraminifera OTUs

Our findings reveal considerable phylogenetic diversity among a limited number of freshwater foraminifera OTUs (Figure 2). We identify several clades and single branches of OTUs with no close relatives, a few OTUs with close relatives to freshwater and terrestrial foraminifera environmental sequences, and even fewer OTUs closely related to well‐documented freshwater foraminiferal species. We acknowledge that at least some of these OTUs we report could be contamination from marine studies in our lab or PCR artifacts, but this final set of 38 OTUs survived our stringent curation steps (e.g., both bioinformatic and hand‐curation) and the phylogenetic position of our OTUs among monothalamid foraminifera suggests that these may indeed be previously uncharacterized freshwater lineages. However, we caution readers not to make strong inferences about OTUs that fall sister to *Allogromia* sp. (e.g., OTU26, OTU42, OTU64, OTU268, OTU304, and OTU423; Figure 2) as we noticed that this reference and these OTUs lack a large portion of information in the hypervariable region we amplified.

Previous analyses of freshwater foraminifera describe five different clades that each represent at least one independent origin from marine lineages (e.g., Holzmann et al. 2021; Siemensma and Holzmann 2023). Our study adds additional environmental sequences to four established freshwater clades: Group 1 (Velomentofexidae), Group 2 (Reticulomyxidae), Group 3 (Lacogromiidae), and Group 4, the largest freshwater group consisting of the Astroperuliidae, Lieberkuehniidae, and Limnogromiidae families (Figure 2). We do not find any close relatives to the group represented by the Edaphoallogromiidae family (see caveat in previous paragraph as it relates to the *Allogromia* sp. reference), which was first observed as a terrestrial lineage (Meisterfeld et al. 2001). Aside from these five groups, we have several OTUs that form “novel” clades that could represent at least four additional independent colonization events (i.e., from marine to freshwater).

### 4.3 Ecological Patterns Among Freshwater Foraminifera Lineages

We provide an example of a focused sampling effort in three distinct low‐pH bog habitats, targeting different ecological sites within each field location. We hypothesized that the pitcher plant samples would be distinct from one another given their founder effect‐like nature and their ability to ecologically filter their microcosm‐like environment (Gilbert et al. 2020). However, we found that only one of the six pitcher plant samples was distinctly different from all other pitcher samples. This finding could be attributed to the fact that we detected few lineages overall, so there are a fewer number of OTUs that would make the pitchers distinct from one another. Alternatively, it is possible that the freshwater foraminifera communities in these pitchers are homogenous, which contrasts the high heterogeneity observed among other protist communities (i.e., ciliates, chrysophytes, Cercozoa) in *Sarracenia* pitcher plants sampled from Hawley and Acadia in the past (Sleith and Katz 2022).

We found no strong patterns indicating that pH was a controlling factor in the occurrence of freshwater foraminifera across ecological sites (Figure S3), suggesting that freshwater foraminifera are more tolerant to varying and low‐pH conditions, as opposed to their marine counterparts. In marine environments, foraminifera assemblages are strongly affected by varying pH (Sen Gupta 2003; Humphreys et al. 2018; Dong et al. 2020; Weinmann et al. 2021), especially those that build calcium carbonate shells which weaken and dissolve in low‐pH environments. Non‐calcareous foraminifera, either “naked” or those with organic shells, are considered among the most able to make the transition into such freshwater environments (Holzmann et al. 2021) as they are not limited by the ability to maintain a calcium carbonate shell in freshwater environments.

One of the most interesting findings of our study was the near‐complete lack of overlap between OTUs found in the greenhouse and in the field locations sampled. Overall, the greenhouse is a long‐term stable environment as opposed to the seasonally fluctuating field sites. We presume that OTUs identified in the field may be capable of dealing with wider fluctuations of abiotic factors than those found in the greenhouse, consistent with the partitioning we see (Figure 4). Furthermore, the greenhouse itself is a unique environment that harbors a curated collection of diverse, exotic, and agricultural flora that likely contributes to the differing freshwater foraminifera community present within this location. In addition, within the greenhouse, we were specifically interested in sampling the different substrates (i.e., wooden versus concrete walls) given that different substrates harbor different microbial communities (Ragon et al. 2012; Risse‐Buhl et al. 2012), which in turn could affect the assemblage of freshwater foraminifera communities present. This avenue is worth exploring further to investigate how different substrate sources may influence the abundance and richness of these relatively rare organisms.

### 4.4 Synthesis

Our study provides a foundational assessment of freshwater foraminifera from several low‐pH bogs, known as extreme environments due to their acidic and nutrient‐poor nature, and from a greenhouse habitat. Through establishing a baseline of diversity and richness among freshwater foraminifera in the northeast United States, we emphasize a key finding: the majority of the richness we characterize is represented by lineages that presently remain molecularly undefined (Figure 2). With increased sampling and the expanding use of molecular techniques coupled with morphological observations, the nature of these clades can be further elucidated and these rare but global organisms can be better understood.

## Author Contributions

**Adri K. Grow:** conceptualization (equal), data curation (lead), formal analysis (equal), investigation (lead), methodology (equal), project administration (equal), software (lead), validation (equal), visualization (lead), writing – original draft (lead), writing – review and editing (lead). **Laura A. Katz:** conceptualization (equal), data curation (supporting), formal analysis (equal), funding acquisition (lead), investigation (supporting), methodology (equal), project administration (equal), resources (lead), supervision (lead), validation (equal), visualization (supporting), writing – original draft (supporting), writing – review and editing (supporting).

## Conflicts of Interest

The authors declare no conflicts of interest.

## Supporting information


Figure S1.
Figure S2.
Figure S3.

File S1.

File S2.

Table S1.
Table S2.
Table S3.
Table S4.
Table S5.
Table S6.
Table S7.
Table S8.
Table S9.
Table S10.

## Data Availability

All raw reads for samples included in these analyses are available under BioProject number PRJNA1108657. Raw reads for failed samples that were removed from this study can be found at the following FigShare repository: https://doi.org/10.6084/m9.figshare.28138985.
