# Supplementary material for: Freshwater Foraminifera Biodiversity in New England (USA): Evaluation of Field Sites and a Botanical Garden
Source: Ecol Evol. 2025 Jul 3;15(7):e71557. doi: 10.1002/ece3.71557 (PMC12223408; doi:10.1002/ece3.71557)
Supplement: Supplementary file 1 — Figure S1. Figure S2. Figure S3. [file ECE3-15-e71557-s001.docx]

**Supplemental information for:** Freshwater Foraminifera Biodiversity in New England (USA): Evaluation of Field Sites and a Botanical Garden

**Authors:** Adri K. Grow and Laura A. Katz

**Corresponding author:** lkatz@smith.edu

Table of Contents:

| **Figure S1** | Images of the two freshwater pools sampled from the Smith College Lyman Plant House and Conservatory. |
| --- | --- |
| **Figure S2** | A phylogenetic tree of a hand-curated broad Foraminifera clade. |
| **Figure S3** | PCoA plot by samples with sample pH information. |


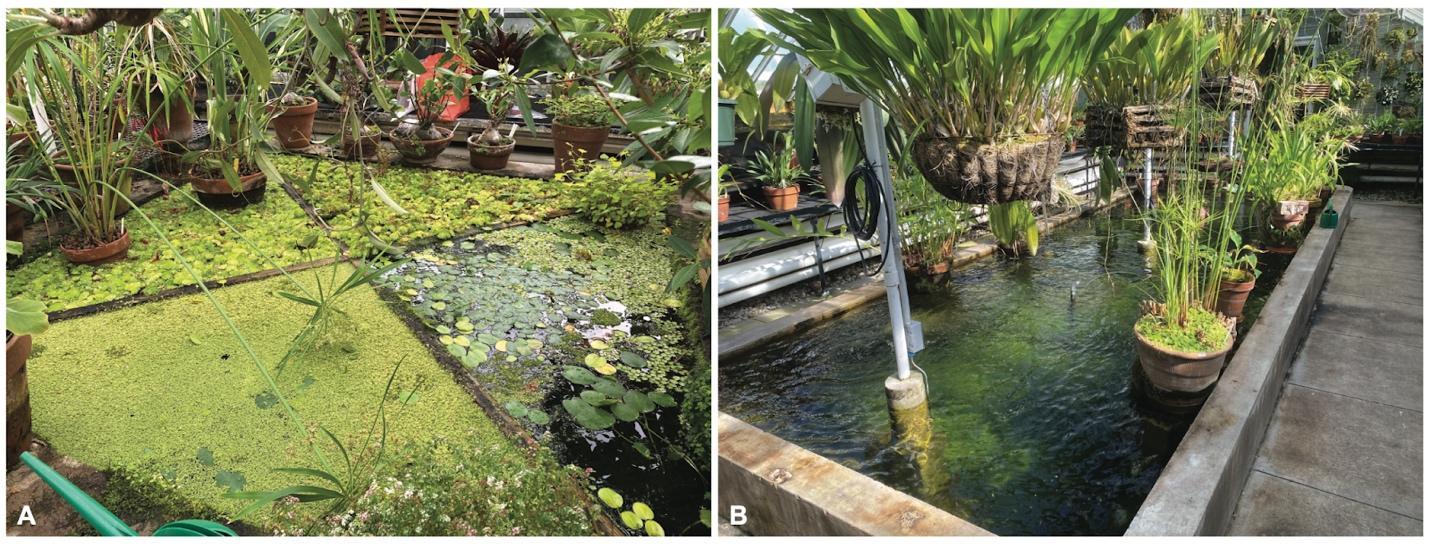


**Figure S1.** Images of the two freshwater pools sampled from in the Smith College Lyman Plant House and Conservatory (42.318903 N, 72.640044 W) on March 1^st^, 2021 and October 23rd, 2023. (A) The 2.5 x 2.5 m concrete pool inside the Warm Temperate House containing Duckweed (*Lemna minor*) and Giant Salvinia (*Salvinia molesta*) separated by a wooden wall. (B) The 2.5 x 6.5 m pool inside the Stove House containing marginal and economically important (e.g. rice) potted flora.


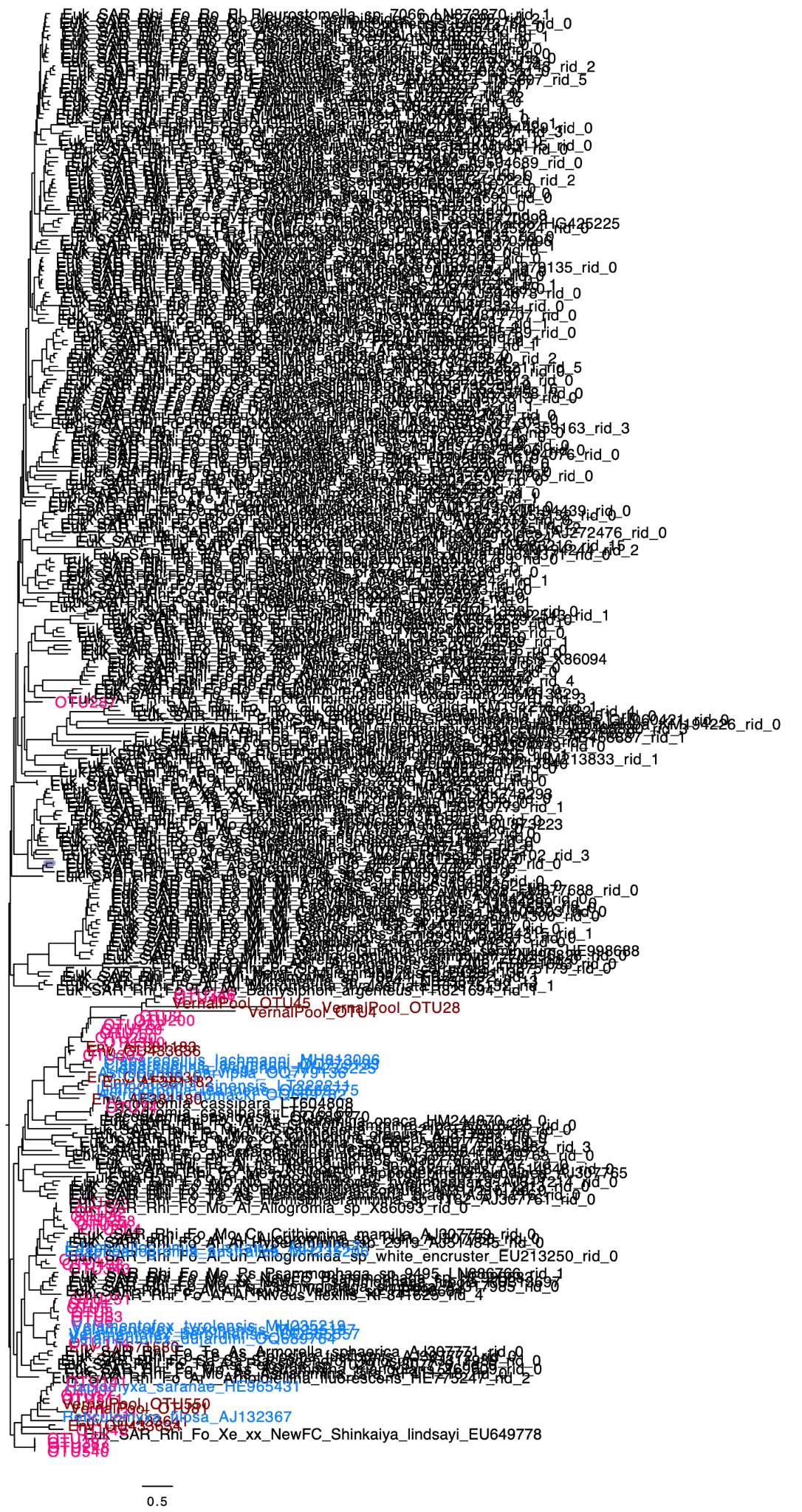


**Figure S2.** A foraminifera phylogenetic tree showing with 262 full-length 18S rDNA references from GenBank (in black) with our putative 38 freshwater foraminifera OTUs in pink. In blue are the 18 freshwater foraminifera full-length references obtained from GenBank in addition to 13 environmental amplicon sequences from either GenBank or Thakur et al. 2022 in brown. Here we show the breadth of the foraminiferal clade and where our OTUs fall among this set of references. The one OTU that is near the middle of the tree is OTU287, which is on a single branch in our main figure and has no close relatives. This OTU should be interpreted with caution.

**
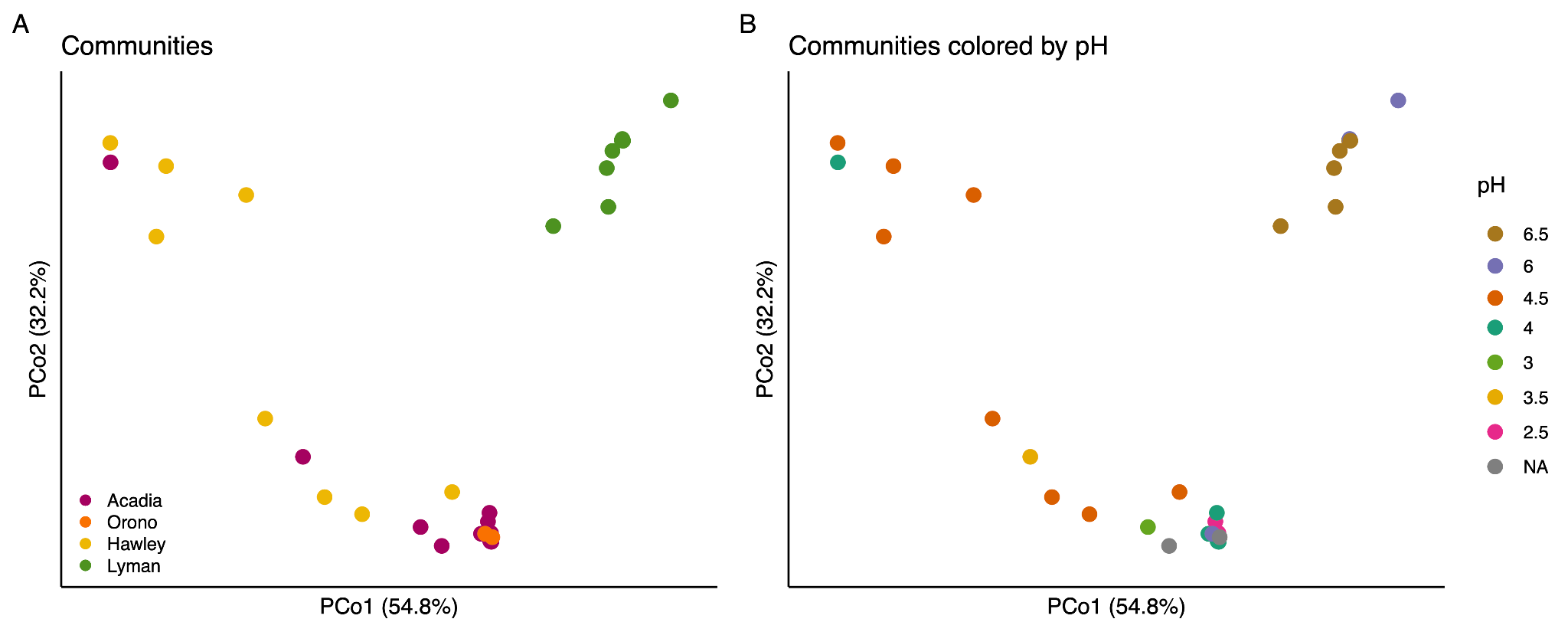
**

**Figure S3.** (A) Principal coordinate analysis (PCoA) with weighted (accounting for relative abundance) UniFrac distance (accounting for phylogenetic relationships) describes 87.0% of variance among samples from the four sampling locations. (B) The same samples as in panel A but colored by pH value in the same ordination space. A pH value of NA means that we did not get a pH measurement for the given site due to a lack of moisture for the pH probe. Here, we see that samples from the greenhouse have a higher pH overall compared to the field sites, but we note that many samples with differing pH overlap on the lower middle portion of the plot. We also see that despite all Hawley samples being the same pH there is no clustering as they are spread widely across PC1 and PC2. We conclude that pH alone is not enough in this case to draw any substantial conclusions. Acadia, Orono, and Hawley = Field locations; Lyman = Greenhouse location. OTU = Operational taxonomic unit.
