# Supplementary figures and images for: Freshwater Foraminifera Biodiversity in New England (USA): Evaluation of Field Sites and a Botanical Garden
Source: Ecol Evol. 2025 Jul 3;15(7):e71557. doi: 10.1002/ece3.71557 (PMC12223408; doi:10.1002/ece3.71557)

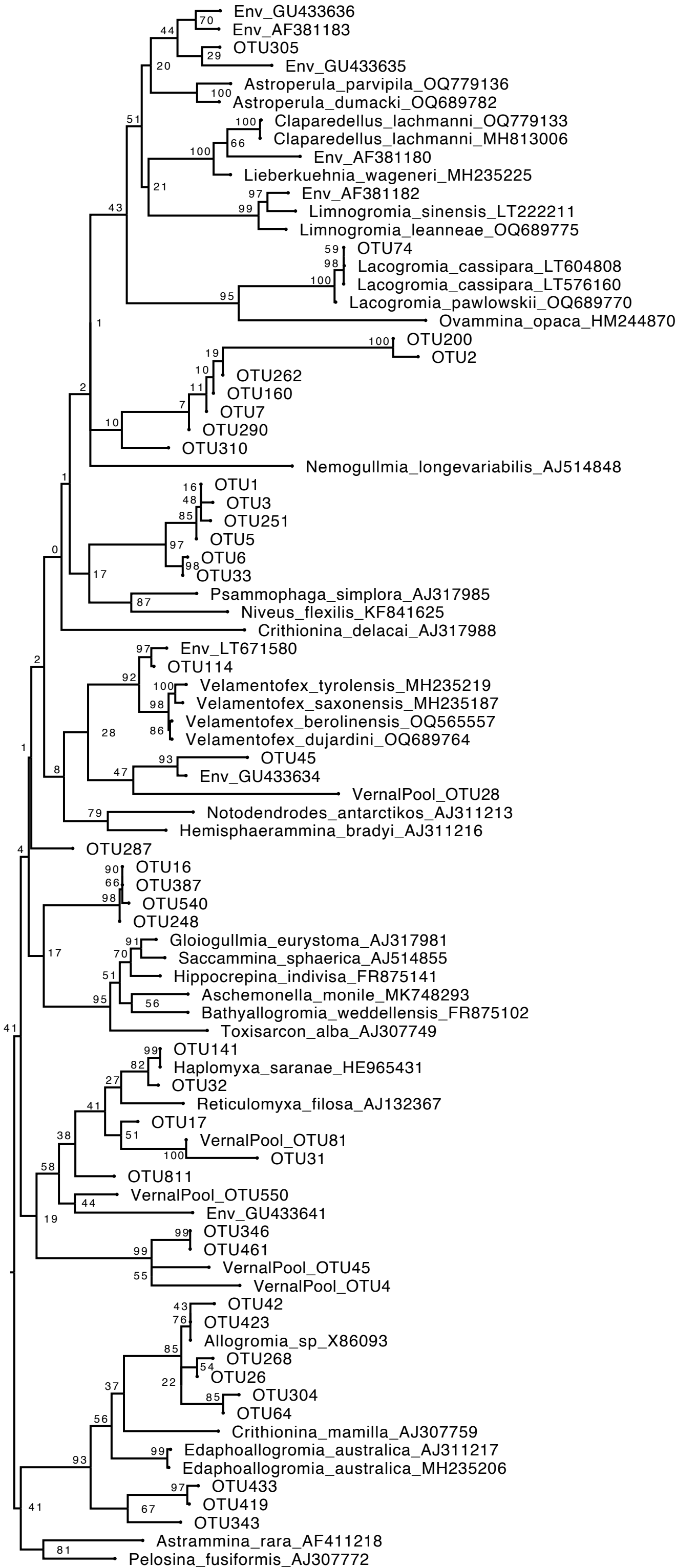

0.3

Supplement: Supplementary file 3 — File S2. [file ECE3-15-e71557-s002.pdf]
